# Correlates of Engagement and Associations With Outcomes in a Cannabis Harm-Reduction Mobile App for Youth With First-Episode Psychosis: Exploratory Analysis of the CHAMPS Pilot Randomized Controlled Trial

**DOI:** 10.2196/84836

**Published:** 2026-08-04

**Authors:** Amani Mahroug, Pamela Lachance-Touchette, Stéphanie Coronado-Montoya, Amal Abdel-Baki, José Côté, Candice Crocker, David Neil Crockford, Jean-Gabriel Daneault, Simon Dubreucq, Maxime Dussault-Laurendeau, Benedikt Fischer, Tania Lecomte, Sophie L’Heureux, Clairélaine Ouellet-Plamondon, Marc-André Roy, Ovidiu Tatar, Philip G Tibbo, Marie Villeneuve, Anne Wittevrongel, Didier Jutras-Aswad

**Affiliations:** 1 Department of Psychiatry and Addiction, Faculty of Medicine, Université de Montréal, Montréal, QC, Canada; 2 Research Centre, Centre Hospitalier de l’Université de Montréal, 900 St Denis Street, Montréal, QC, H2X 1P1, Canada, 1 514 890 8000 ext 35703, 1 514 412 7320; 3 Department of Psychiatry, Centre Hospitalier de l’Université de Montréal, Montréal, QC, Canada; 4 Faculty of Nursing, Université de Montréal, Montréal, QC, Canada; 5 Department of Psychiatry, Dalhousie University, Halifax, NS, Canada; 6 Department of Psychiatry, Cumming School of Medicine, University of Calgary, Calgary, AB, Canada; 7 Hotchkiss Brain Institute, University of Calgary, Calgary, AB, Canada; 8 Hôpital en santé mentale Albert-Prévost, Montréal, QC, Canada; 9 Hôpital Charles Lemoyne, Longueuil, QC, Canada; 10 Centre for Applied Research in Addiction and Mental Health, Simon Fraser University, Vancouver, BC, Canada; 11 Research and Graduate Studies Division, University of the Fraser Valley, Abbotsford, BC, Canada; 12 Department of Psychiatry, University of Toronto, Toronto, ON, Canada; 13 School of Population Health, University of Auckland, Auckland, New Zealand; 14 Department of Psychiatry, Federal University of Sao Paulo, Sao Paulo, Brazil; 15 Department of Psychology, University of Montréal, Montréal, QC, Canada; 16 Centre de Recherche de l'Institut Universitaire en Santé Mentale de Montréal, Montréal, QC, Canada; 17 Centre Intégré Universitaire de Soins et Services Sociaux de la Capitale Nationale, Institut Universitaire en Santé Mentale, Clinique Notre-Dame des Victoires, Québec City, QC, Canada; 18 Department of Psychiatry and Neurosciences, Laval University, Laval, QC, Canada; 19 Centre de Recherche de l'Institut Universitaire en Santé Mentale de Québec, Québec City, QC, Canada; 20 Centre de Recherche CERVO, Québec City, QC, Canada; 21 University Institute on Addictions, Montréal, QC, Canada

**Keywords:** cannabis, harm reduction, psychosocial intervention, first-episode psychosis, digital intervention, engagement, adherence, mHealth, mobile health, pilot trial, randomized controlled trial

## Abstract

**Background:**

Continued cannabis use among young people with first-episode psychosis (FEP) has been linked to poorer clinical and functional outcomes (eg, increased symptom severity and higher relapse rates). Digital harm-reduction interventions may represent a promising, person-centered approach to reduce at-risk cannabis use behaviors in this population. However, evidence remains limited regarding which subgroups are more likely to engage with these interventions and whether specific levels of engagement are required to achieve more favorable cannabis-related outcomes.

**Objective:**

This exploratory, hypothesis-generating analysis of the CHAMPS (Cannabis Harm-Reducing App to Manage Practices Safely) pilot randomized controlled trial (RCT) evaluated a cannabis harm-reduction mobile app for youth with FEP in early intervention services (EIS). The objectives of this study were to assess engagement by examining associations between selected sociodemographic factors and module completion and to determine whether achieving specific completion thresholds was associated with improvements in cannabis-related outcomes.

**Methods:**

Cannabis-related outcomes (Marijuana Problems Scale [MPS], Protective Behavioral Strategies for Marijuana [PBSM] scores, and days of cannabis use) were self-assessed at baseline and at week 6 (primary end point), week 12, and week 18 (postrandomization). Participants were categorized into low to moderate (0‐4 modules) and high (5‐6 modules) completion groups, and selected sociodemographic factors were compared between groups using bivariate analyses. Mixed-effects models, adjusted for baseline values and the covariates sex and cannabis use disorder (CUD) status, were fitted to evaluate whether module thresholds were associated with improvements in cannabis-related outcomes.

**Results:**

Data from 96 participants, including 46 in the CHAMPS+EIS arm and 50 in the EIS-only arm (1:1 ratio), were analyzed under a modified intention-to-treat principle. Individuals in the high-engagement group reported higher baseline social support and higher educational level (*P*=.005 and *P*=.01). No statistically significant associations were observed between specific module completion thresholds and cannabis-related outcomes in adjusted mixed-effects models.

**Conclusions:**

Participants with higher social support and higher education were more likely to engage with CHAMPS. Although descriptive analyses suggested a potential gradient of improvement with increasing module completion, no specific completion threshold was robustly associated with improvements in outcomes. Given the multifactorial nature of engagement, supporting subgroups at risk of lower app use and examining metrics beyond module completion may enhance the impact of CHAMPS. These findings are hypothesis-generating, given their exploratory nature, and require replication in a future efficacy trial.

## Introduction

Across Canada, cannabis use is highly prevalent among young adults. Although cannabis use is generally associated with low health risk in the general population, specific consumption behaviors (eg, frequency, mode of administration), individual factors (eg, age, genetic vulnerability), and societal factors (eg, normalization of cannabis use) increase the likelihood of negative health outcomes [1,2]. One subgroup of particular concern is youth experiencing a first episode of psychosis (FEP), who show a significantly higher prevalence of cannabis use disorder (CUD; 42%‐53%) [1,2] compared to the general population (about 3%) [3,4]. Continued cannabis use while receiving early intervention services (EIS) for psychosis is associated with increased symptom severity, higher relapse rates, more frequent hospitalizations, and reduced functional outcomes [1,3,5].

Although abstinence is often recommended as the ideal treatment target, many young users are ambivalent or unwilling to quit cannabis completely, limiting their engagement and treatment outcomes [6]. Notably, few face-to-face interventions aimed at abstinence or reduction have had significant effects on abstinence or reduction rates in young people with psychotic disorders and CUD [7,8]. Harm-reduction approaches, which aim to mitigate substance use–related harms without requiring abstinence, offer a pragmatic and person-centered alternative that is progressively being recommended by public health authorities, clinicians, and people with lived experience [9-12]. The Lower-Risk Cannabis Use Guidelines (LRCUG), recently adapted to address psychosis-related risks (LRCUG-PSYCH), provide a foundation for evidence-based harm reduction but have yet to be operationalized into scalable interventions [13,14]. Digital health interventions may help address this gap by offering flexible and accessible support that can be integrated into users’ daily lives [15-18]. However, findings from recent reviews suggest mixed effectiveness of digital interventions for cannabis use among youth, partly due to heterogeneity in intervention design, targeted behaviors, and user engagement [19,20].

Recent literature emphasizes engagement as a critical determinant of successful digital mental health interventions. Engagement (defined as the extent to which users interact with the intervention) has also been conceptualized as a multidimensional construct influenced not only by the intervention itself but also by the context of use, the mechanisms of action behind the digital tool, and the targeted behavior that work together [21-23]. Common indicators include uptake (ie, downloading and using the intervention at least once), sustained use (ie, continued activity over time), and adherence or completion (ie, following the intervention as intended) [24]. Understanding and clarifying how users interact with these tools and what drives sustained use is essential, while taking into account that these metrics may capture related but distinct aspects of intervention use and therefore require cautious interpretation [24]. Some studies also suggest that individual-level factors such as gender, education, and digital literacy may influence how users interact with and benefit from digital tools [25,26]. In particular, baseline social support might be relevant to engagement, as individuals with higher interpersonal (relational and peer) support may have more resources, incentives, and/or stability to initiate and sustain their participation in digital interventions [27]. Conversely, supportive features embedded within digital interventions (eg, peer interaction, built-in buddy features, e-coaching, etc) have also been described as strategies associated with greater use and engagement [28].

Several frameworks guide the development of digital health tools to foster engagement [29]. One of them, the Behavior Change Wheel (BCW), addresses key psychosocial determinants (capability, opportunity, and motivation), while the person-based model [30] emphasizes designs that reflect the most lived experience by incorporating user feedback [31,32]. Given these frameworks and the research gap, our team developed the app, CHAMPS (Cannabis Harm-Reducing App to Manage Practices Safely). CHAMPS is a self-guided, digital psychosocial intervention designed for young adults with FEP that integrates motivational interviewing approaches, harm-reduction principles, and skills training, and is designed to be incorporated within EIS [33]. Engagement features embedded in CHAMPS include push notifications to complete unfinished modules, personalized reflective questions related to chosen SMART (Specific, Measurable, Achievable, Relevant, Time-based) goals, links to video content, and an automated booster session 4 weeks postintervention. Findings from the pilot randomized controlled trial (RCT) demonstrated its acceptability and feasibility within this context [34].

Building on this initial study and to scale up CHAMPS for broader implementation, it is important to identify which specific subgroups benefit most from the intervention [35] and what is the minimum level of engagement associated with benefits. As highlighted by Haller et al [36], analyzing predictors and moderators can offer insights into for whom and under what circumstances internet-based interventions are most likely to be favorable, guiding intervention tailoring to support a better patient-treatment fit [37]. Moreover, only a limited number of studies have examined engagement correlates and trajectories in this context [38,39]. Therefore, to address these gaps, our objective was to assess engagement by examining associations between selected sociodemographic factors (gender, ethnicity, education, and social support) and module completion and to determine whether achieving specific completion thresholds was associated with improvements in cannabis-related outcomes.

## Methods

### Study Design and Randomization

Our study presents a secondary analysis of the CHAMPS trial, a multicenter, 2-arm, parallel-group, pilot RCT. This pilot trial compared the cannabis harm-reduction e-intervention, CHAMPS, in conjunction with EIS (CHAMPS+EIS), to the standalone EIS in young adults with FEP who use cannabis. CHAMPS was co-designed with people with lived experience through a patient advisory committee [40]. Consenting participants were randomly allocated in a 1:1 ratio to the intervention or control group, using a digital, stratified, permuted block design with varying block sizes implemented by the CRCHUM data management team. Randomization was stratified by CUD status and sex. Because of the nature of the intervention, the research staff and clinicians involved in data collection were unblinded; however, the research staff conducting the analyses remained blinded.

### Ethical Considerations

This study trial is reported in accordance with the CONSORT-EHEALTH (Consolidated Standards of Reporting Trials of Electronic and Mobile Health Applications and Online Telehealth), version 1.6.1, guidelines (Checklist 1) and was registered prospectively at ClinicalTrials.gov (NCT04968275) in July 2021 [41]. The detailed protocol was described by Coronado-Montoya et al [40], and the pilot feasibility and acceptability analysis was published by our team [34]. CHAMPS was conducted in accordance with the Good Clinical Practice (GCP) guidelines and the Declaration of Helsinki, alongside Canadian standards (Tri-Council Policy Statement and Health Canada Division 5) and applicable provincial and institutional ethical guidelines (20.433). Approval for the protocol was obtained from the local research ethics boards (REBs) at each participating site. The study started in December 2021 and was completed in October 2023. No major changes were made to the protocol after trial initiation, other than adding 2 new sites.

Informed consent was obtained from each participant prior to enrollment, and the informed consent form was approved by each REB, allowing for secondary analysis without additional consent. All participant-related information, including case report forms, assessments, and reports, was kept strictly confidential, and participants were identified only by means of a numeric study identifier specific to each participant. All computerized databases identified participants using numeric codes and were password-protected. The link between participants’ identification numbers and their names was kept in a password-protected document accessible only to research staff, and the document is stored on the CRCHUM secure server for a duration of 10 years. No identification of individual participants is possible from any figures or supplementary materials presented in this manuscript. Participants were compensated CAD $30 (equivalent to US $21) per visit and CAD $150 (equivalent to US $106) in total for completing the study.

### Participants and Recruitment

Young adults with FEP who were using cannabis and were open to changing their cannabis use practices (N=101) were recruited into the study through referrals from EIS psychiatrists or case managers. Recruitment took place at 6 specialized EIS sites for the treatment of early psychosis in Canada and is described in Coronado-Montoya et al [40]. Inclusion criteria included being 18 to 35 years old, having a diagnosis of any psychotic disorder according to *DSM-5* (*Diagnostic and Statistical Manual of Mental Disorders*, Fifth Edition) criteria, being followed for a minimum of 3 months at an early psychosis clinic, using cannabis (at least once in the past month), being interested in changing cannabis-related practices, and understanding French or English. Exclusion criteria were concurrent enrollment in another cannabis-related intervention or current pursuit of treatment for CUD aimed at reducing or ceasing cannabis consumption. Participants identified as seeking treatment for CUD were instead directed to a concurrent clinical trial [42] or to usual clinical care. Digital literacy was not assessed. Individuals were considered eligible if they met all the inclusion criteria and none of the exclusion criteria. Eligible participants were randomly assigned (in a 1:1 ratio) to either the intervention arm (n=51) or the EIS-only arm (n=50). Sample size justification is described elsewhere [40]. In this exploratory analysis, given that 5 participants (intervention arm, n=5) never started the intervention, data from the remaining 96 participants were used in a modified intention-to-treat (mITT) analysis since participants who did not initiate the intervention could not contribute to post-initiation data needed to estimate changes in outcomes over time [43].

### Study Intervention

Participants accessed the CHAMPS e-intervention through a mobile health (mHealth) application and completed 6 psychotherapeutic, fully automated online modules within 6 weeks, followed by an automated booster session 4 weeks after the final module. Each module was designed to be completed in less than 10 minutes. Participants were guided to reflect on their cannabis use practices and their impact on their lives (modules 1 and 2). Module 3 provided information on harm-reduction strategies, while module 4 taught goal-setting skills. Modules 4 to 6 helped participants set personalized harm-reduction–related goals. The booster module guided participants in reviewing the main components of CHAMPS, such as exploration of their cannabis use and their goal setting. Additional resources were available, including evidence-based articles and videos cocreated with individuals with lived experience. All participants received EIS. The complete study procedures, including detailed module content, have been reported elsewhere [40]. The pilot RCT lasted up to 22 weeks in total, including a 14-day screening period, an additional 14-day window for baseline assessments, a 6-week intervention period plus a 4-week period to complete the booster module, and a 4-week follow-up period. Follow-up assessments were conducted by research assistants at weeks 12 and 18.

### Outcomes and Measures

#### Cannabis-Related Outcomes

Use of evidence-based strategies that promote safer cannabis experiences was assessed using the Short Form Protective Behavioral Strategies for Marijuana (PBSM) measure, a 17-item self-report questionnaire with strong psychometric properties [44]. Increases in PBSM scores reflect improvements in protective behavioral strategies related to cannabis use. Changes in cannabis-related problems over time were measured using the Marijuana Problems Scale (MPS), a 19-item self-report instrument [45,46]. Decreases in MPS scores indicate fewer cannabis-related problems over time. The number of days of cannabis use during the past 2 weeks was assessed using the Timeline Follow-Back (TLFB), a self-report measure with high test-retest reliability that has been validated against other measures of drug-related problems and in clinical populations [47].

#### Sociodemographic and Clinical Characteristics

Gender, educational level, ethnicity or race, social support, symptom severity, CUD status, and cannabis consumption group were assessed at baseline. Social support was assessed using the Social Provisions Scale-10 (SPS-10) [48], and symptom severity was assessed using the Positive and Negative Syndrome Scale (PANSS-6) [49]; neither variable was dichotomized. Gender was categorized into 3 groups: “men,” “women,” and “other” (including transgender and nonbinary individuals). Educational level was dichotomized into “secondary school not completed’’ and “secondary or more” (diploma or certificate from a trade school or vocational program, university undergraduate degree, and other [eg, CEGEP, Collège d'enseignement général et professionnel]). Ethnicity or race was dichotomized into “White” and “individuals of other racial and ethnic backgrounds,” and CUD status was dichotomized into “none to mild” and “moderate to severe.” Cannabis consumption group at baseline was dichotomized into “higher frequency” (using a minimum of 5 days per week according to the TLFB at baseline) and “lower frequency” (using a maximum of 4 days per week according to the TLFB at baseline).

#### Engagement Measures

Module completion was summarized descriptively into 2 engagement groups: “low to moderate” (0‐4 modules) and “high” (5‐6 modules). To evaluate how many modules were needed to observe an impact on changes in outcomes in the CHAMPS+EIS arm, we used module completion thresholds (ie, completion of at least 1 module to at least 6 modules) as a predictor in linear regression. Module completion was chosen because it is a pragmatic, objective proxy for engagement that quantitatively reflects the extent to which participants completed the intervention [50-52].

### Adverse Events

Information on adverse events was collected electronically by study staff and assessed systematically. No serious adverse events were reported.

### Statistical Analysis

#### Descriptive and Baseline Analysis

Baseline demographic and clinical characteristics were summarized by study arm and for the overall sample. Main outcomes (MPS total score, PBSM total score, and days of cannabis use) were described at every time point (baseline, week 6, week 12, and week 18). Selected baseline demographics (social support, education, ethnicity, and cannabis use frequency) were also described across both engagement groups (low to moderate vs high). These variables were selected based on prior literature, suggesting that they might be potentially relevant to engagement in online interventions for psychosis or cannabis use [19,25]. Associations between baseline characteristics and engagement groups were assessed with Wilcoxon rank-sum tests for continuous variables and Fisher exact tests for categorical variables. Continuous variables were reported as mean (SD) or median (IQR) based on the normality of their distributions and categorical variables as frequencies and percentages. Analyses of the differences between the engagement groups were conducted using a Bonferroni-adjusted α level of .0125 per test (.05/4).

Linear mixed-effects models (LMMs) were fitted to evaluate changes in the continuous outcomes (PBSM total score and MPS total score), while generalized mixed-effects models (GLMMs) were fitted to evaluate changes in the non-normally distributed outcomes (days of cannabis use) across study time points. For each model, the primary outcome was treated as the dependent variable, while the study arms, time points, and their interaction were modeled as fixed effects. Participants nested within site were included as a random effect to account for within-site and within-participant correlations. All models were adjusted for the stratification variables, sex and CUD status, and for each baseline outcome. These analyses were specified a priori to provide an overview of outcomes trajectories across the trial. To evaluate whether engagement (module completion thresholds) predicted improvements in the main outcomes at week 6, linear regression analyses were conducted only in the CHAMPS+EIS intervention arm, adjusting for sex, CUD status, and baseline outcome values. Consistent with prior literature stating that patterns between engagement outcomes are not always linear, exploring if a potential minimum engagement was associated with outcomes improvement was hypothesis-generating only [21]. For each main outcome, separate linear regression models were fitted within the CHAMPS+EIS arm for each separate module completion threshold. Model outputs included β coefficients and *P* values and were conducted using a Bonferroni-adjusted α level of .008 per test (.05/6). All analyses were conducted using the lme4 package in R (version 4.3.2; R Core Team) [53].

#### Sensitivity Analysis

Module completion was also modeled as a continuous predictor of outcome changes at week 6 to assess whether the results were consistent when engagement was modeled as a dose-like measure rather than a threshold-based exposure.

#### Missing Data

All available observations were included in our analyses with maximum likelihood estimation. No imputation method was performed given the exploratory nature of the analyses, with the assumption that data were missing at random (MAR).

## Results

### Participant Characteristics

**Figure 1.**
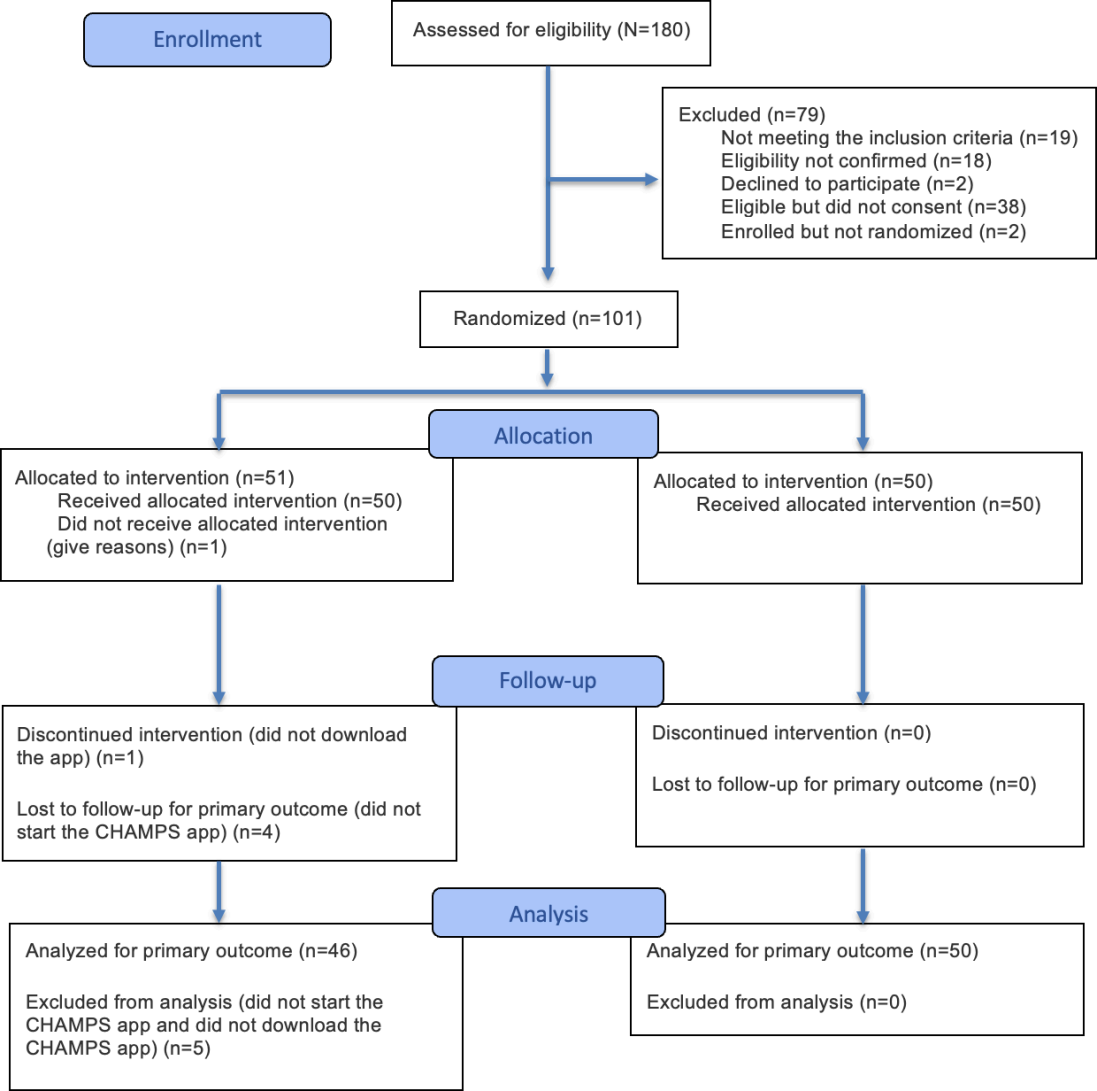
CONSORT (Consolidated Standards of Reporting Trials) flowchart of the CHAMPS pilot study (n=96). CHAMPS: Cannabis Harm-Reducing App to Manage Practices Safely.

A total of 101 participants were randomized to either the intervention CHAMPS+EIS or the control group, EIS only. Given that 5 participants never started the intervention, data from the remaining 96 participants were used in our mITT analysis, as stated in the CONSORT flow diagram [41] (Figure 1).

Table 1 summarizes baseline demographics and clinical characteristics of the participants. On average, participants were 25 (SD 3.9) years old, and most had completed at least a secondary high school diploma (n=69, 72%). Participants were predominantly men (n=69, 72%), White (n=57, 59%), and reported using cannabis at least 5 days per week (n=74, 77%). Although most baseline characteristics were balanced across study arms, the overall sample distribution was asymmetric for a few variables, with a greater proportion of participants reporting postsecondary education, being White, a higher frequency of cannabis use, and men gender.

Selected baseline sociodemographic characteristics across engagement groups—low to moderate (n=18) and high (n=28)—are described in Table 2. Individuals with high engagement reported higher levels of baseline social support compared with those with low-to-moderate engagement (median 33.5, IQR 31.5-38.0) vs (median 28.5, IQR 27.3-32.8; *P*=.005). A greater proportion of participants in the high engagement group also had a higher educational level (23/28, 82% vs 8/18, 44%; *P*=.01). Other sociodemographic factors did not differ significantly between the groups after correction for multiple comparisons.

**Table 1.** Baseline sociodemographic and clinical characteristics of the included participants^a^.

Variable at baseline		CHAMPS^b^+EIS^c^ (n=46**)**	Control (n=50)	Total (N=96)
Age (years), mean (SD)		25.3 (4.0)	24.8 (3.8)	25.0 (3.9)
Sex, n (%)				
Male		36 (78)	39 (78)	75 (78)
Female		10 (22)	11 (22)	21 (22)
Gender, n (%)				
Men		33 (72)	36 (72)	69 (72)
Women		8 (17)	10 (20)	18 (19)
Other (transgender, nonbinary, no answer)		5 (11)	4 (8)	9 (9)
Education level^d^, n (%)				
Secondary not completed		15 (33)	12 (24)	27 (28)
Secondary or more		31 (67)	38 (76)	69 (72)
Ethnicity or race^e^, n (%)				
White		32 (70)	25 (50)	57 (59)
Other racial and ethnic people		19 (41)	31 (62)	50 (52)
Social support, median^f^ (IQR)		32.5 (29.3‐36.0)	31.5 (27.5‐36.0)	32 (28.8‐36.0)
Symptom severity, mean^g^ (SD)		11 (5.6)	10.5 (5.7)	10.7 (5.6)
Cannabis use disorder^h^ (CUD), n (%)				
No CUD (0-1) to mild CUD (2-3)		23 (50)	26 (52)	49 (51)
Moderate CUD (4-5) to severe CUD (≥6)		23 (50)	24 (48)	47 (49)
Cannabis consumption group, n (%)				
Higher frequency (≥5 days per week)		32 (70)	42 (84)	74 (77)
Lower frequency (<5 days per week)		14 (30)	8 (16)	22 (23)
Cannabis consumption days, mean (SD)		9.2 (5.3)	9.6 (5.4)	9.4 (5.3)

a Categorical variables are reported as n (%); continuous variables are reported as mean (SD) or median (IQR).

b CHAMPS: Cannabis Harm-Reducing App to Manage Practices Safely.

c EIS: early intervention services.

d Educational level was dichotomized as “Secondary not completed” (elementary school or lower, or some secondary school partially completed) and “Secondary or more” (secondary school diploma, diploma or certificate from a trade school or vocational program, university undergraduate degree, or other (eg, CEGEP [Collège d'enseignement général et professionnel]).

e Participants could select more than one ethnic group, the percentages do not add up to 100%.

f Social support was assessed with the Social Provisions Scale-10 (SPS-10).

g Symptom severity was assessed with the Positive and Negative Syndrome Scale (PANSS-6).

h CUD subcategory numbers (0‐1, 2‐3, 4‐5, and ≥6) reflect the number of symptoms in the *DSM-5 *(*Diagnostic and Statistical Manual of Mental Disorders*, Fifth Edition).

**Table 2.** Participant baseline profiles by engagement level in the CHAMPS^a^+EIS^b^ arm only^c^.

Variable at baseline	Engagement levels		*P* value
	Low to moderate (0‐4 modules; n=18)	High (5‐6 modules; n=28)	
Education level, n (%)			.01^d^
Secondary not completed	10 (56)	5 (18)	
Secondary or more	8 (44)	23 (82)	
Ethnicity or race^e^, n (%)			.04
White	10 (56)	22 (79)	
Other racial and ethnic people	12 (66)	7 (25)	
Social support, median (IQR)	28.5 (27.2-32.8)	33.5 (31.5-38.0)	.005^d^
Cannabis consumption group, n (%)			.19
Higher frequency	15 (83)	17 (61)	
Lower frequency	3 (17)	11 (39)	

a CHAMPS: Cannabis Harm-Reducing App to Manage Practices Safely.

b EIS: early intervention services.

c Categorical variables are reported as n (%); continuous variables as median (IQR). Group comparisons at baseline were conducted across 2 engagement levels (low to moderate and high) using Fisher exact test for categorical variables and Wilcoxon rank-sum tests for continuous variables.

d Statistically significant after Bonferroni correction for 4 comparisons (adjusted α=.0125).

e Participants could select more than one ethnic group, the percentages do not add up to 100%.

### Changes in Main Outcomes Across Study Time Points

**Figure 2.**
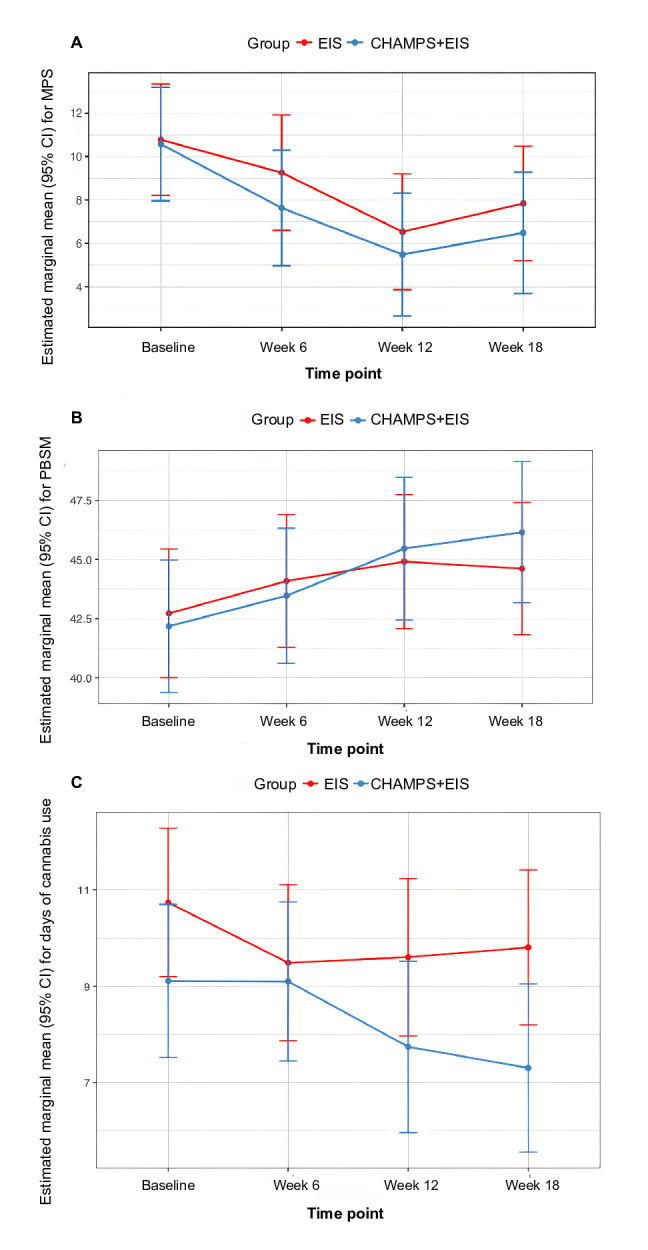
(A) Adjusted Marijuana Problems Scale (MPS) score, (B) Protective Behavioral Strategies for Marijuana (PBSM) score and (C) days of cannabis use trajectory. All figures represent estimated marginal means with 95% CI by study group and assessment time. CHAMPS: Cannabis Harm-Reducing App to Manage Practices Safely; EIS: early intervention services.

Table S1 in Multimedia Appendix 1 summarizes MPS scores, PBSM total scores, and days of reported cannabis consumption in the last 14 days, with corresponding trajectories depicted in Figure 2. Descriptive data, estimated marginal means (EMMs), and adjusted mean differences from the LMM are reported in Tables S1 and S2 Multimedia Appendix 1. Findings suggest overall lower MPS scores in the CHAMPS+EIS group at all follow-up time points, although these differences did not reach statistical significance (Tables S1 and S2 in Multimedia Appendix 1). For instance, at week 6, the adjusted mean difference in MPS between the CHAMPS+EIS arm and the control arm was 1.4 (95% CI −3.8 to 1.0), with similar trends at week 12 and week 18.

### Impact of Module Completion on Main Outcomes in the CHAMPS+EIS Arm

**Figure 3.**
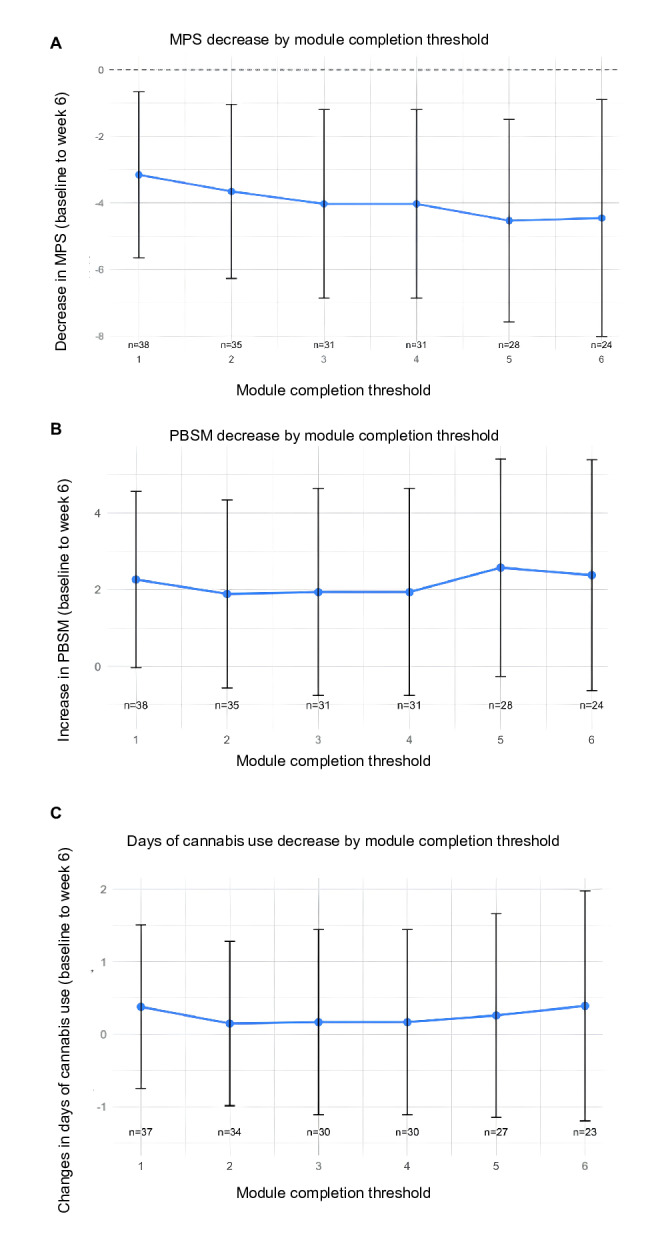
Mean changes from baseline to week 6 in (A) Marijuana-related Problems Scale (MPS), (B) Protective Behavioral Strategies for Marijuana (PBSM), and (C) days of cannabis use, according to module completion threshold. Analyses included only participants from the Cannabis Harm-Reducing App to Manage Practices Safely (CHAMPS)+early intervention services (EIS) arm. Sample sizes (n) for each threshold are indicated below the *x*-axis. Error bars represent 95% CIs.

Linear regression analyses within CHAMPS+EIS found no statistically significant associations between module completion thresholds (ranging from ≥1 to ≥6 modules) and any primary outcome at week 6 after adjusting for the baseline outcome, sex, and CUD status (Table 3). Although the differences were not statistically significant, descriptive analyses indicated greater improvements among participants completing 5 or more modules for the MPS and PBSM outcomes (Figure 3). Sensitivity analyses showed findings in the same direction when the number of modules was treated as a continuous predictor (Table S3 in Multimedia Appendix 1).

**Table 3.** Linear mixed-effects models and generalized mixed-effects models of module completion thresholds and changes in primary outcomes at week 6 among the Cannabis Harm-Reducing App to Manage Practices Safely (CHAMPS)+early intervention services (EIS) group only^a^.

Outcome and predictor	β coefficient (95% CI)	SE	*P* value
MPS^b^ score (modules)			
≥1	−0.7 (−7.6 to 6.2)	3.5	.84
≥2	0.14 (−5.2 to 5.4)	2.7	.96
≥3	−1.5 (−5.8 to 2.8)	2.2	.48
≥4	−1.5 (−5.8 to 2.8)	2.2	.48
≥5	−2.3 (−6.2 to 1.6)	2	.26
≥6	−2 (−5.7 to 1.7)	1.9	.32
PBSM^c^ score (modules)			
≥1	9.76 (1.2 to 18.4)	4.4	.03^d^
≥2	2.26 (−4.4 to 9)	3.4	.51
≥3	1.37 (−4 to 6.8)	2.8	.63
≥4	1.37 (−4.3 to 7.1)	2.9	.63
≥5	3 (−2.1 to 8)	2.6	.26
≥6	1.6 (−3.3 to 6.5)	2.5	.52
Days of cannabis use (modules)			
≥1	3.9 (−1.6 to 9.4)	2.8	.18
≥2	0.31 (−3.4 to 4)	1.9	.87
≥3	−0.02 (−3.1 to 3.1)	1.6	.99
≥4	−0.02 (−3.1 to 3.1)	1.6	.99
≥5	−0.15 (−2.9 to 2.6)	1.4	.92
≥6	0.23 (−2.5 to 2.9)	1.4	.87

a Estimates represent adjusted associations between module completion thresholds and changes in outcomes from baseline to week 6. Each row reflects a separate model for each module completion threshold and main outcome, adjusting for multiple comparisons. All models were adjusted for sex, cannabis use disorder status, and baseline score for each outcome.

b MPS: Marijuana Problems Scale.

c PBSM: Protective Behavioral Strategies for Marijuana.

d Statistically significant after Bonferroni correction for 6 comparisons (adjusted α=.008).

## Discussion

### Principal Findings

In this exploratory pilot study, we examined which sociodemographic factors were associated with engagement and whether specific engagement thresholds were associated with improved cannabis-related outcomes in our digital health intervention, CHAMPS, among young individuals with FEP. Overall, participants in the CHAMPS+EIS group who completed more than 5 modules were more likely to have higher social support and higher educational levels at baseline than those who completed fewer modules. Interestingly, while descriptive analyses indicated a potential gradient of improvement with increasing module completion, no specific module completion threshold was found to be significantly associated with improved cannabis-related outcomes at the study end point. Taken together, these findings suggest that engagement with CHAMPS occurs across diverse profiles but is likely to be influenced by social support and education, and that setting a specific minimum number of completed modules may not capture the variability in how individuals derive benefit or not.

Although CHAMPS previously demonstrated overall acceptability and feasibility across a diverse sample [34], participants with more module completion (5 modules or more) tended to report greater baseline social support, a finding that aligns with the existing literature on digital mental health interventions. Higher social support (be it from clinicians or peers) is a well-documented facilitator of positive adherence to self-guided interventions by reinforcing motivation and accountability [21,23,24,54]. In other studies, individuals with higher baseline social support were also described as more prone to engage with the social elements of digital interventions, and therefore with the digital intervention itself [23]. Similarly, higher education has been repeatedly associated with increased engagement, as educational attainment could facilitate not only comprehension of psychoeducational content but also the internalization and application of harm-reduction strategies [55]. However, digital interventions, such as those delivered in self-guided formats, may require a certain level of cognitive engagement and autonomous learning, which can pose a barrier to responsiveness in the context of FEP where cognitive impairment is a core feature for many individuals [56,57]. Although the present digital intervention took this into account when designing the app, a self-guided intervention like CHAMPS may represent an additional barrier to intervention responsiveness in people with lower educational levels. Individuals with lower educational attainment may benefit from additional guided support while emphasizing the need of tailoring content to different literacy levels, including digital literacy and health literacy [58].

Although our linear regression with the predictor of module completion threshold in the intervention arm only did not yield significant results, visual inspection showed a potential gradient of improvement with increasing module completion. As described by Perski et al [23,59], this observation is consistent with the literature on digital intervention research that describes a dose-response link between higher engagement levels and optimal response. The CHAMPS intervention included personalized feedback, psychoeducational techniques, and the evidence-based use of protective strategies [40]. Participants engaged in goal-setting tasks in modules 4 and 6, and were guided to set personalized harm reduction–related goals between modules 4 and 6. These observations highlight the need to consider robust in-app strategies, such as reminders, involvement of clinicians, and other support, to help users reach a selected threshold of module or content and thereby maximize the benefits of the intervention [28].

Drawing on the theoretical foundations of the BCW [32], CHAMPS underscored the interplay among capability, opportunity, and motivation. Our results may also reflect that adopting harm-reduction behaviors is an engaging process and behavioral engagement that requires more than mere exposure to the intervention; it also involves active effort and internalization [60]. In this sense, engagement is not a passive process but one that may be facilitated by baseline factors such as those described in this study [23]. Digital intervention research has also shown that merely sharing content with individuals is not enough to change their behavior. The timing and method of delivery play an important role in this [61]. A sequential and cumulative module structure, in which each module builds upon previous information while introducing more strategies, could be hindered by incomplete engagement. As emphasized in recent digital intervention literature, engagement is not a unidimensional construct but is rather shaped by the interplay among contextual elements, diverse components (behavioral, cognitive, and affective), individual factors, and the design of the intervention [23,54,60 ]. This may explain the heterogeneous effects observed across some subgroups in our research and suggests that simple usage metrics may provide only an incomplete representation of engagement [62].

Finally, this pilot trial was not powered to test the efficacy of CHAMPS in EIS, and the intervention arm did not demonstrate statistically significant differences in cannabis-related outcomes compared with the control group. However, descriptive data showed consistently lower scores on the cannabis-related problems outcome (MPS score) by about 2 points, which could be considered clinically relevant, at all follow-up time points in the CHAMPS+EIS group [63]. These differences, although not statistically significant, warrant further investigation in a properly powered efficacy trial to formally test CHAMPS’s potential benefits in this population.

While the present study was underpowered to fully test complex 3-way interactions, findings suggest that some subgroups could require higher levels of exposure to achieve comparable outcomes, but this may only capture a facet of a more complex engagement process. Qualitative interviews conducted in our pilot trial are expected to provide more nuanced insights into engagement motives, facilitators, and barriers that cannot be captured by objective usage data alone [64]. Together, these points further extend the growing call in the literature for personalizing digital interventions and avoiding “one-size-fits-all” delivery models [65].

### Limitations

Several limitations should be considered. First, the modest sample size limited the generalizability of the findings and did not allow for a formal efficacy analysis. Second, the mixed-model analyses yielded mostly nonsignificant values, and the reported difference in social support between engagement groups was not adjusted for potential confounding variables (eg, education level, racial or ethnic composition), both reflecting limited statistical power. Third, as expected for a pilot trial, some attrition occurred, with progressively fewer participants completing each follow-up assessment. This loss-to-follow-up may reflect diverse factors, including low cannabis problem severity at baseline (participants with low MPS and high PBSM scores at baseline may perceive limited relevance of the intervention), digital fatigue, technical barriers, and app usability issues. This differential attrition could have introduced retention bias, as those who remained in the study may have differed from dropouts, potentially underestimating or overestimating engagement and related outcomes. Finally, additional bias may stem from data missing not at random, for example, if participants who responded more poorly were more likely not to complete some assessments and/or to disengage from the digital intervention.

### Conclusions

In this sample of youth with FEP in EIS care, most individuals who had higher social support and a higher educational level engaged more with our digital cannabis harm-reduction intervention. No specific completion threshold was robustly associated with improvements in outcomes, suggesting that engagement is a multifactorial construct that goes beyond quantitative metrics (module completion). Interpretation of the findings should remain cautious, given their exploratory nature, the number of comparisons performed, and the possibility that some groups may have been underrepresented. Given the high prevalence of cannabis use among individuals with FEP, expanding and enhancing existing interventions could help mitigate the individual and societal impact of psychosis while easing the workload of clinicians in EIS. Further research, including a large-scale efficacy trial and qualitative methods, could explore whether refinements such as social support features (eg, family or peer prompts and/or in-app reminders, adapted and flexible support from clinicians), engagement-boosting strategies (eg, closer clinician follow-up, motivating content, etc), and literacy-tailored supports (eg, guided journal prompts, clinician support, etc) would improve intervention uptake through engagement and result in more favorable cannabis-related outcomes.

## Supplementary material

10.2196/84836 Multimedia Appendix 1 Descriptive statistics and regression analyses for the CHAMPS pilot study.

10.2196/84836 Checklist 1 CONSORT-EHEALTH checklist (V 1.6.1).

## Abbreviations

BCW: Behavioral Change Wheel
CEGEP: Collège d'enseignement général et professionnel
CHAMPS: Cannabis Harm-Reducing App to Manage Practices Safely
CONSORT-EHEALTH: Consolidated Standards of Reporting Trials of Electronic and Mobile Health Applications and Online Telehealth
CUD: cannabis use disorder
*DSM-5*: *Diagnostic and Statistical Manual of Mental Disorders*, Fifth Edition
EIS: early intervention services
FEP: first-episode psychosis
GCP: Good Clinical Practice
GLMM: generalized mixed-effects model
LMM: linear mixed-effects model
LRCUG: Lower-Risk Cannabis Use Guidelines
MAR: missing at random
mHealth: mobile health
mITT: modified intention-to-treat
MPS: Marijuana Problems Scale
PANSS-6: Positive and Negative Syndrome Scale
PBSM: Protective Behavioral Strategies for Marijuana
RCT: randomized controlled trial
REB: research ethic board
SMART: Specific, Measurable, Achievable, Relevant, Time-based goals
SPS-10: Social Provisions Scale-10
TLFB: Timeline Follow-Back
