# Supplementary material for: Correlates of Engagement and Associations With Outcomes in a Cannabis Harm-Reduction Mobile App for Youth With First-Episode Psychosis: Exploratory Analysis of the CHAMPS Pilot Randomized Controlled Trial
Source: JMIR Form Res. 2026 Aug 4;10:e84836. doi: 10.2196/84836 (PMC13436797; doi:10.2196/84836)
Supplement: Supplementary file 2 [file formative-v10-e84836-s001.docx]

**Supplementary Tables**

**Table S1.** Descriptive results of main outcomes throughout the CHAMPS pilot study.

**Table S2.** Linear mixed-effects models and generalized-mixed effects models for MPS, PBSM and days of cannabis use by treatment group and timepoint.

**Table S3.** Linear mixed-effects models and generalized-mixed effects models of module completion (continuous) and changes in primary outcomes at week 6 among CHAMPS+EIS group only.

**Table S1. Descriptive results of main outcomes throughout the CHAMPS pilot study.**

| **Outcome** | **Timepoint** | **CHAMPS + EIS**  **(n=46)** | **Control (EIS)**  **(n=50)** | **Total**  **(N=96)** |
| --- | --- | --- | --- | --- |
| **MPS, mean (SD)** | | | | |
|  | Baseline | (46) 10.7 (7.1) | (50) 10.8 (8.2) | (96) 10.7 (7.6) |
|  | Week 6 | (41) 7.8 (6) | (40) 9 (7.4) | (81) 8.4 (6.7) |
|  | Week 12 | (29) 4.7 (4.6) | (39) 6.9 (7.2) | (68) 6 (6.3) |
|  | Week 18 | (31) 6 (5.3) | (42) 8.2 (8.3) | (73) 7.3 (7.3) |
| **PBSM, mean (SD)** | | | | |
|  | Baseline | (46) 42.2 (8) | (50) 42.7 (8.3) | (96) 42.5 (8.2) |
|  | Week 6 | (41) 43.1 (10.8) | (40) 43.6 (6.7) | (81) 43.3 (9) |
|  | Week 12 | (29) 46.2 (11.5) | (39) 44.6 (6.9) | (68) 45.3 (9.1) |
|  | Week 18 | (31) 45.7 (10.3) | (42) 44.0 (8.2) | (73) 44.8 (9.2) |
| **Days of cannabis use, mean (SD)** | | | | |
|  | Baseline | (46) 9.1 (5.3) | (50) 10.7 (4.4) | (96) 9.95 (4.9) |
|  | Week 6 | (39) 9.2 (5.3) | (40) 9.55 (5.4) | (79) 9.4 (5.3) |
|  | Week 12 | (29) 7.9 (5.5) | (39) 9.49 (5) | (68) 8.8 (5.2) |
|  | Week 18 | (31) 7.8 (5.5) | (42) 9.7 (5.30) | (73) 8.9 (5.4) |

Continuous variables are reported as (n), mean (SD). Abbreviations: MPS = Marijuana Problems Scale, PBSM = Protective Behavioral Strategies for Marijuana.

**Table S2. Linear mixed-effects models and generalized-mixed effects models for MPS, PBSM and days of cannabis use by treatment group and timepoint.**

| **Outcome** | **Timepoint** | **Estimated marginal means (SE)** | | **Adjusted mean difference (95% CI)** | **Interaction test**  **(type III, *F*_df_, *P)*** |
| --- | --- | --- | --- | --- | --- |
|  |  | **CHAMPS (CHAMPS+EIS) (n=46)** | **Control**  **(EIS) (n=51)** |  |  |
| **MPS** | | | | | |
|  | Baseline | 11 (0.9) | 11.1 (0.8) | -0.03 (-2.3 to 2.2) | *F*_3,233_= 0.4, *P*=.8 |
|  | Week 6 | 8.1 (0.9) | 9.5 (0.9) | -1.4 (-3.8 to 1) |  |
|  | Week 12 | 5.8 (1.1) | 6.8 (0.9) | -1 (-3.6 to 1.6) |  |
|  | Week 18 | 6.9 (1) | 8.2 (0.9) | -1.3 (-3.8 to 1.3) |  |
| **PBSM** |  |  |  |  |  |
|  | Baseline | 42.5 (0.9) | 42.5 (0.9) | -0.1 (-2 to 2.2) | F_3,234_= 0.8, *P*=.5 |
|  | Week 6 | 43.9 (0.9) | 44.2 (0.9) | -0.3 (-2.7 to 2.2) |  |
|  | Week 12 | 46 (1) | 45 (0.9) | 0.8 (-1.8; 3.4) |  |
|  | Week 18 | 46.4 (1) | 44.6 (0.9) | 1.8 (-0.8; 4.4) |  |
| **Days of cannabis use** | | | | | |
|  | Baseline | 9.8 (0.6) | 10.1 (0.5) | -0.4 (-1.8 to1.1) | F_3,234_= 1.3, *P*= .3 |
|  | Week 6 | 9.8 (0.6) | 8.9 (0.6) | 1 (-0.6 to 2.6) |  |
|  | Week 12 | 8.5 (0.7) | 8.9 (0.6) | -0.4 (-2 to 1.3) |  |
|  | Week 18 | 8.1 (0.7) | 9.1 (0.6) | -1 (-2.7 to 0.7) |  |

Linear mixed-effects models for repeated measures (LMMs) and generalized-mixed effects models for repeated measures (GLMMs) were fitted to baseline-adjusted outcomes. Models included fixed effects for treatment group, timepoint, and their interaction, random intercepts for participants nested within site, and covariates (sex, CUD). Adjusted effects are model-adjusted mean differences (CHAMPS – Control) within each timepoint, reported with 95% confidence intervals (CIs). Interaction tests correspond to Type III ANOVA of the Group × Timepoint term. *P*< .0125 was considered statistically significant. Abbreviations: CI: Confidence interval, MPS = Marijuana Problems Scale, PBSM = Protective Behavioral Strategies for Marijuana.

**Table S3. Linear mixed-effects models and generalized-mixed effects models of module completion (continuous) and changes in primary outcomes at week 6 among CHAMPS+EIS group only.**

| **Outcome** | **Predictor** | **β coefficient (SE)** | ***P* value** | **95% CI** |
| --- | --- | --- | --- | --- |
| **MPS score** | | | | |
|  | Modules (continuous) | -0.1 (0.4) | 0.74 | (-0.9 to 0.7) |
| **PBSM score** | | | | |
|  | Modules (continuous) | 0.5 (0.5) | 0.41 | (-0.6 to 1.5) |
| **Days of cannabis use** | | | | |
|  | Modules (continuous) | -0.01 (0.02) | 0.52 | (-0.06 to 0.03) |

Estimates represent adjusted associations between module completion as a continuous predictor and outcome changes from baseline to week 6. All models were adjusted for sex, CUD and baseline score of each outcome. *P*< .017 was considered statistically significant. Abbreviations: MPS = Marijuana Problems Scale, PBSM = Protective Behavioral Strategies for Marijuana.
